# Soil microbiomes conditioned by long‐term warming affect plant belowground performance

**DOI:** 10.1111/plb.70182

**Published:** 2026-01-19

**Authors:** C. Le Noir de Carlan, E. Verbruggen, L. Colaert‐Sentenac, M. Cougnon, P. Sigurðsson, B. D. Sigurdsson, J. Debode, C. De Tender

**Affiliations:** ^1^ Biology Department, Plants and Ecosystems (PLECO) University of Antwerp Antwerp Belgium; ^2^ Department of Environmental Sciences University of Basel Basel Switzerland; ^3^ INRAE, IRHS, Université d'Angers Beaucouzé France; ^4^ Plant Sciences Unit, Flanders Research Institute for Agriculture, Fisheries and Food (ILVO) Merelbeke Belgium; ^5^ Agricultural University of Iceland Borgarnes Iceland; ^6^ Department of Biochemistry and Microbiology Ghent University Ghent Belgium

**Keywords:** Global change, plant biomass, roots, soil microbiome, warming

## Abstract

- Global change affects plant performance, both directly through warming and indirectly through changes in their biotic and abiotic surroundings. Soil microbes can critically influence plant performance, but are vulnerable to warming themselves. Disentangling direct effects of warming on plants from those intermediated by changes in microbial populations is complex under field conditions.
- To distinguish those effects, we monitored the performance of *Agrostis capillaris* and *Anthoxanthum odoratum* grown under uniform and controlled glasshouse conditions in soils inoculated with soil microbiomes conditioned by ambient, medium (14 years; MTW) or long‐term (>55 years; LTW) geothermal warming. This was replicated under normal watering or drought conditions to additionally assess stress resistance. Furthermore, we analysed the microbiome of the inocula through metabarcoding to identify root‐associated fungi and compare their relative abundance under different warming conditions.
- We found a decreased belowground biomass of both plant species when grown with LTW‐conditioned microbiomes, with an exacerbated effect under drought for *Ag. capillaris*. We did not observe an associated increase in aboveground biomass, resulting in an increased aboveground biomass:belowground biomass ratio. These changes coincided with concurrent increases in the relative abundance of putative plant pathogens and arbuscular mycorrhizal fungi.
- We therefore conclude that soil microbes can mediate warming effects on plant performance through reduced belowground biomass.

## Graphical abstract

Soil microbial communities conditioned by long‐term warming affect the plant performance of *Anthoxantum odoratum* and *Agrostis capillaris* by reducing belowground biomass.

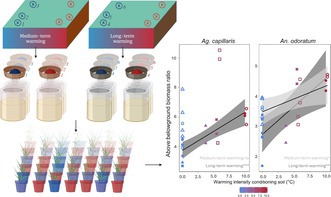

## INTRODUCTION

World temperature has been steadily increasing over the past century, which affects plants' physiology not only directly, but also indirectly by simultaneously altering their biotic and abiotic environment (Classen *et al*. 2015; Crous 2019; IPCC 2021). High‐latitude regions are undergoing climate change more rapidly than other regions (IPCC 2021), where rising temperatures are driving increased decomposition and mineralisation (Chapin *et al*. 1995; Kirschbaum 1995), as well as shifts in plant physiology (Gargallo‐Garriga *et al*. 2017), phenology (Valdés *et al*. 2019), belowground traits (Bhattarai *et al*. 2023) and community composition (Walker *et al*. 2006). While the impact of climate change on plants has primarily been studied in terms of abiotic pressures, changes in other pressures such as pests, pollinators and mutualists caused by climate change have gained rising attention (Gérard *et al*. 2020; Lehmann *et al*. 2020; Magnoli *et al*. 2023). In particular, soil microbes, increasingly recognised as sensitive to environmental changes (Cavicchioli *et al*. 2019), can influence plant growth in a multitude of ways, through interactions ranging from antagonistic to mutualistic (De Vries *et al*. 2023). A change in the microbial community is therefore expected to have profound effects on plant performance.

Specifically, warming‐induced alteration of the soil microbiome can directly affect plant growth. Several studies reported that plant pathogenic fungi benefit from warming, and it is predicted that the proportion of soilborne pathogens will increase globally and in most biomes with elevated temperatures (Geml *et al*. 2015; Semenova *et al*. 2015; Delgado‐Baquerizo *et al*. 2020). On the contrary, most plants benefit from associations with mycorrhizal fungi that enhance nutrient and water uptake in exchange for photosynthetically fixed carbon. In grasslands, plants mostly associate with arbuscular mycorrhizal fungi (AMF) (Smith & Read 2008). While AMF generally tend to benefit from warming (Compant *et al*. 2010), responses are highly context‐dependent (Duarte & Maherali 2022): For instance, AMF were favoured by long‐term soil warming in Icelandic grasslands (Radujkovic *et al*. 2018; Zhang *et al*. 2020), while their richness was greatly decreased by warming in an alpine meadow (Shi *et al*. 2021). Given these potentially strong influences on plants, it is no surprise that studies have also established the influence of soil microbes on plant fitness (Lau & Lennon 2011) or phenology (Wickander *et al*. 2021). While plant‐associated microorganisms could either accelerate or mitigate climate change effects on plants (Sharma *et al*. 2022), microbial community assembly may be disrupted by warmer conditions (Metze *et al*. 2024), potentially altering how they influence plant performance under warming as well. Moreover, if in the short term warming primarily acts as a disturbance that favours some taxa – particularly opportunistic such as fungal plant pathogens – over others, we may expect that in the long‐term plant–soil interactions will stabilise again. Such an overreaction depending on the duration of warming has previously been observed (Melillo *et al*. 2017; Walker *et al*. 2020). These microbe‐mediated effects are however challenging to capture under field conditions and to distinguish from the direct effects of warming.

As the microbial community composition has a profound effect on plant performance, soil health, defined as the continued capacity of soil to function as a vital living ecosystem that sustains plants, animals and humans (Hou 2023), has been a highly studied topic in recent years. While some approaches assess soil health using broad microbiome metrics including microbial diversity (Ferris & Tuomisto 2015) or compositional shifts under stress (Joos *et al*. 2023), an alternative approach relies on investigating the proportion of specific microbial groups such as antagonistic (*e.g*. plant pathogens) or symbiotic (*e.g*. AMF) microbes used as indicators for their putative effect on plant performance (Nielsen & Winding 2002).

Here, we tested the effect of soil microbiomes conditioned by (1) soil warming and (2) different warming durations on plant biomass in a controlled greenhouse experiment. For this, we made use of the unique ForHot site in Southwest Iceland (Sigurdsson *et al*. 2016) where we collected soils that were either unwarmed, geothermally warmed for 14 years or geothermally warmed for over 55 years, subjected to different warming intensities but maintained at constant warming levels with natural seasonal fluctuations caused by geothermal activity (see Sigurdsson *et al*. 2016 for details). These soils were used to separately grow two dominant grass species at the ForHot site, *Agrostis capillaris* L. and *Anthoxanthum odoratum* L. in mesocosms. We expected (1) warming to shift the soil microbiome towards a higher proportion of plant pathogens and AMF, reducing plant biomass because of both potential infection (pathogens) and lowered reliance on plant roots for nutrient acquisition (AMF). Furthermore, (2) we expected more pronounced differences between plants grown in unwarmed soils and soils conditioned by warming under MTW compared to LTW. Lastly, we investigated the effect of drought stress on plant performance in relation to warming‐induced soil microbiomes. We expected (3) soils conditioned by warming to experience a further decline in health, resulting in an even lower plant performance compared to unwarmed soils.

## MATERIALS AND METHODS

### Soil sampling and experimental set‐up

Soils originated from the ForHot experimental site, located in the Southwest of Iceland near Hveragerdi (64.008° N, 21.178° W, Sigurdsson *et al*. 2016). This site consists of two unmanaged grasslands located 2–2.5 km apart on geothermal gradients where soils have been naturally warmed since either 2008, after an earthquake (MTW) or over 55 years (LTW). In each grassland, several ~35‐m‐long transects covering a wide range of similar warming intensities ranging from unwarmed (*i.e*. ambient) to +10 °C were established perpendicularly to the soil temperature gradients to ensure spatial distance between replicate warming levels is maximised and uniform, with soil temperature continuously monitored at 10 cm depth since 2012. The similar vegetation and soil properties make the sites well suited for comparing the effect of microbiomes conditioned by short‐ and long‐term soil warming on plant performance.

After snowmelt, on 13 April 2022, we collected both unwarmed and warmed soils from three different transects in each site. As we targeted the topsoil with similar warming intensities across sites (MTW and LTW), we were limited by the amount of soil available for sampling, as the soil is shallow and the temperature gradient tends to be fairly steep causing small patches with similar temperatures. We therefore had to allow for some temperature variation in our soil collection with warming intensities ultimately ranging from +3.7 to +10 °C compared to the soil temperature of the unwarmed plot of the same transect (Table S1). For each warming condition (*i.e*. ambient or warmed within MTW or LTW), we sampled three different locations. The soil samples were kept and regularly mixed at room temperature for 3 days to remove excess water while ensuring they would not dry out (Joos *et al*. 2023) and subsequently stored at 9 °C until the beginning of the greenhouse experiment on the 22 April 2022. We manually removed all plant residuals and mixed each soil with sterilised sand (1:1 v/v). The soil samples were incubated in the greenhouse for 14 days, with a day/night light regime of 10/14 h and an approximate temperature of 22 °C by day and 18 °C at night. Air humidity was *ca*. 80% throughout the experiment (Melle, Belgium). We then further mixed the soils (‘inocula’) with sterile sand (1:1 v/v) for a final proportion of 25:75 (soil:sand) to have a comparable soil texture across treatments. To account for potential differences in the nutrient contents of the soils, we added slow‐release fertilisers (Basacote, Compo expert: 16% N, 8% P and 12% K) to a concentration of 3.5 g/L of soil following manufacturer instructions in each mesocosm.

**Fig. 1:**
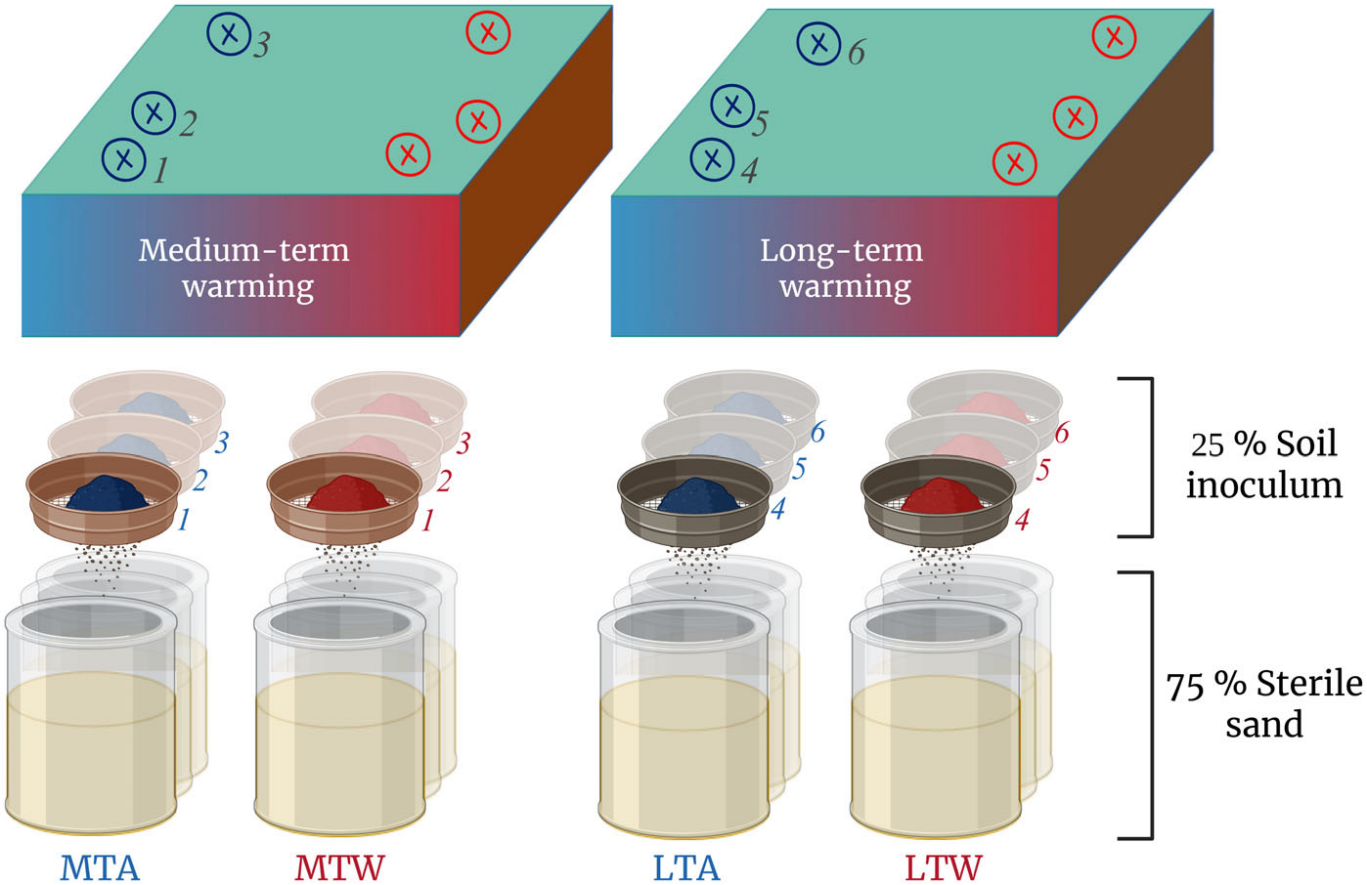
Experimental set‐up for growing media preparation: soil was sampled from the ForHot experimental site, from grasslands with either medium‐term (14 years; MT) or long‐term (>55 years; LT) warming. In each grassland, three transects (referred to as replicates 1–3) where soils were either ambient (A) or warmed (W) were sampled. Any remaining root material was manually removed (hereby represented by the sieve). Soils were used to inoculate a growing media (sterile sand) following a 25:75 ratio. The numbers represent the transects in which both ambient and warmed soils were collected.

We surface sterilised *An*. *odoratum* and *Ag*. *capillaris* seeds (Cruydt Hoeck, Nijeberkoop, NL) using 5% sodium hypochlorite for 2 min and subsequently 70% ethanol for 5 min. We rinsed them in several sterile water baths and put them on filter paper for germination in a 100% humidity chamber for 9 days in the case of *An. odoratum* and 7 days for *Ag. capillaris*. Each mesocosm consisted of 15 cm × 13 cm (diameter × height) sterilised pots filled with 1,300 ml of the previously prepared sand/soil mixture (Fig. 1). The bottom of each pot was amended with a sterilised plastic filter to avoid substrate losses but allow for water drainage. We transplanted five seedlings of similar size in each mesocosm that contained one of the three replicate soils from either ambient or warmed locations in either the LTW or MTW site (Fig. 1), following a pentagonal shape to avoid imbalanced competition and edge effects. Two mesocosms were set up per soil sample as technical replicates to enhance the reliability of measurements. Hence, per plant species this resulted in 2 warming levels (ambient/warmed) × 2 durations (LTW × MTW) × 3 biological replicate soils × 2 technical duplicate mesocosms = 24 mesocosms. For both plant species, one extra set of 24 mesocosms was subjected to a drought treatment and the other served as a control, thus resulting in a total of 96 mesocosms randomly placed and mixed throughout the whole experiment. Soil water content at field capacity was measured at the beginning of the experiment. The first 2 weeks, all mesocosms were uniformly watered to keep soils moist and favour successful seedling growth, and afterwards they were watered every *ca*. 4 days. After 57 days, half of the plants were no longer watered for 14 days while the others (*i.e*. controls) were ample‐watered, to a moisture content of 40–60%. Plants from all treatments were then clipped at 5 cm, and drought‐treated mesocosms were rewatered for another 3 weeks to allow recovery, after which all were harvested: Aboveground biomass was carefully clipped at soil level. All roots were sampled and thoroughly washed. We oven‐dried all plant material at 70 °C for *ca*. 48 h for biomass analyses.

### Soil fungal analyses

To investigate the proportions of fungal putative plant pathogens and AMF in the different inocula, we used a microbial community dataset of soil samples collected on the same locations in April 2021, after snowmelt, 1 year before the sampling campaign for the present study. Briefly, soils were collected from root in‐growth cores that had been installed in October 2019 (experimental set‐up described in Method S1). We are confident that these reflect the microbial composition of the soils used for this study as we found very low variation over time in this site (data not shown). We therefore used these samples to support observations made from our experiment outputs; however, we acknowledge these should be interpreted somewhat cautiously. Briefly, we extracted total DNA using the DNeasy PowerSoil kit (Qiagen, Venlo, NL) and targeted the fungal ITS1 (for putative plant pathogens) region and the V4 region of the 18S rRNA gene (for AMF) for amplicon sequencing on an Illumina MiSeq platform (Illumina Inc., San Diego, CA, USA). Using the DADA2 inference algorithm (Callahan *et al*. 2016), we generated amplicon sequence variants (ASVs) and assigned them to taxonomy using the UNITE database. A detailed protocol can be found in Supporting Information (Method S1).

### Data analyses

All data processing and analyses were performed in R (version 4.0.4., R Core Team 2020). First, we used linear mixed effect models to test the effects of warming under MTW and LTW, under both watered and drought conditions on plant aboveground and belowground biomass measured per mesocosm, setting transect as random. We further analysed the influence of warming intensity on the aboveground biomass:belowground biomass ratio through linear regression models. Second, for the inocula, we identified AMF (members of Glomeromycotina) and putative fungal plant pathogens using the FungalTrait database (Põlme *et al*. 2020) and compared their relative abundance under unwarmed or warmed soils within each transect.

## RESULTS

### Aboveground and belowground biomass

**Fig. 2:**
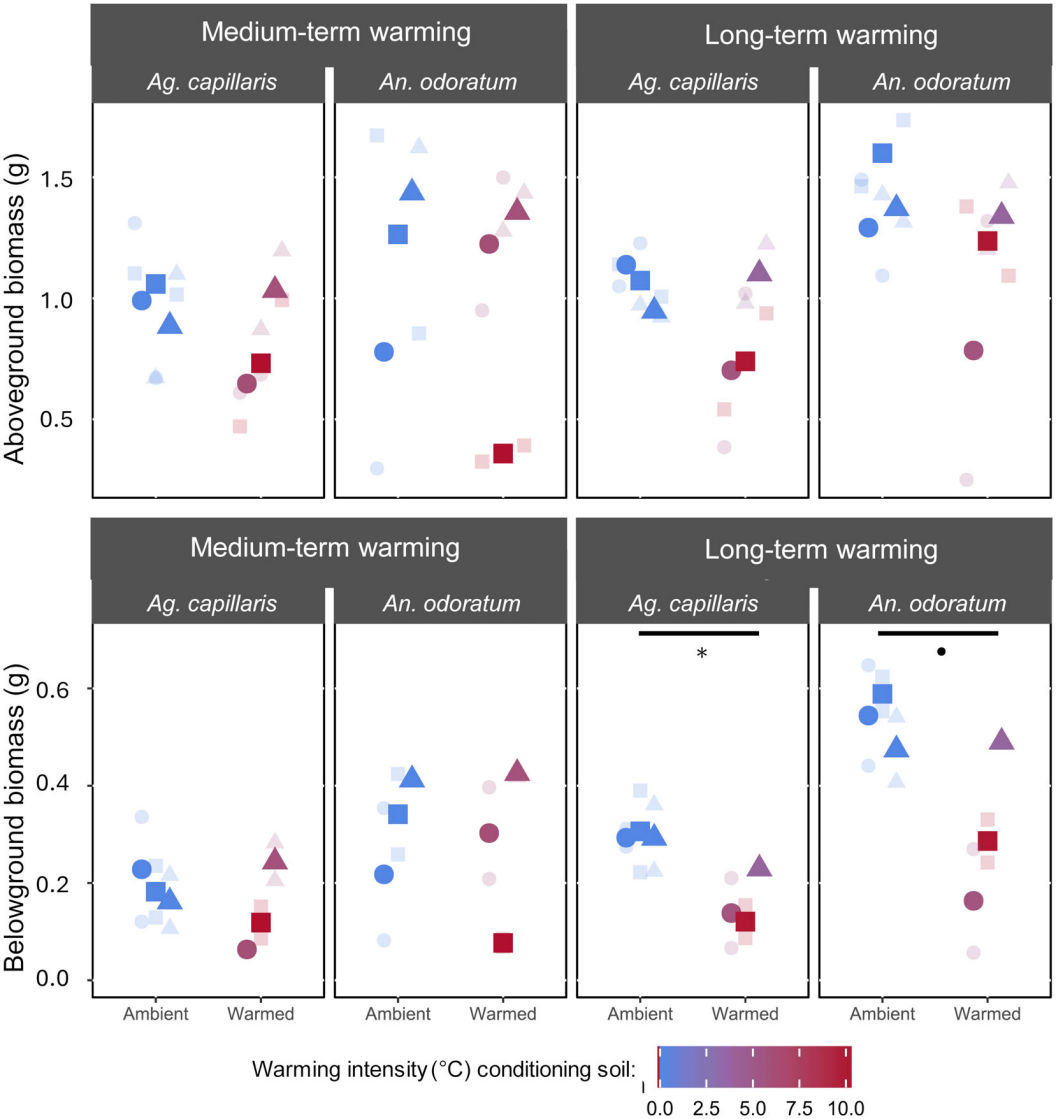
Aboveground (top) and belowground (bottom) dry biomass of *Ag. capillaris* and *An. odoratum* grown under control conditions (*i.e*. watered) in soils conditioned by ambient or warming (colour) for either medium or long term. The bigger and fully coloured points are the averages of technical replicates that are shown in transparent and smaller. Statistics were performed on averages of technical duplicates and only *P* values under 0.1 are shown (*P* < 0.1, *P < 0.05 and **P < 0.01). Shapes indicate that they are from the same transect.

Under non‐drought growth conditions, *Ag. capillaris* revealed lower belowground biomass when grown in soils inoculated by LTW‐conditioned microbiomes (Fig. 2) compared to unwarmed control soil (*P* = 0.02). Although not statistically significant for *An. odoratum* (*P* = 0.09), it suggests a similar trend. We did not observe a change in aboveground biomass (*P* = 0.21 for *Ag. capillaris* and *P* = 0.20 for *An. odoratum*) nor a change in total plant biomass grown in LTW‐conditioned soil microbiomes (Fig. 2; *P* = 0.12 for *Ag. capillaris* and *P* = 0.14 for *An. odoratum*). Both plant species grown in soils inoculated with MTW‐conditioned microbiomes did not show altered aboveground (*P* = 0.24 for *Ag. capillaris* and *P* = 0.65 for *An. odoratum*), belowground (*P* = 0.44 for *Ag. capillaris* and *P* = 0.66 for *An. odoratum*) or total plant biomass (*P* = 0.29 for *Ag. capillaris* and *P* = 0.65 for *An. odoratum*) compared to the unwarmed soils.

**Fig. 3:**
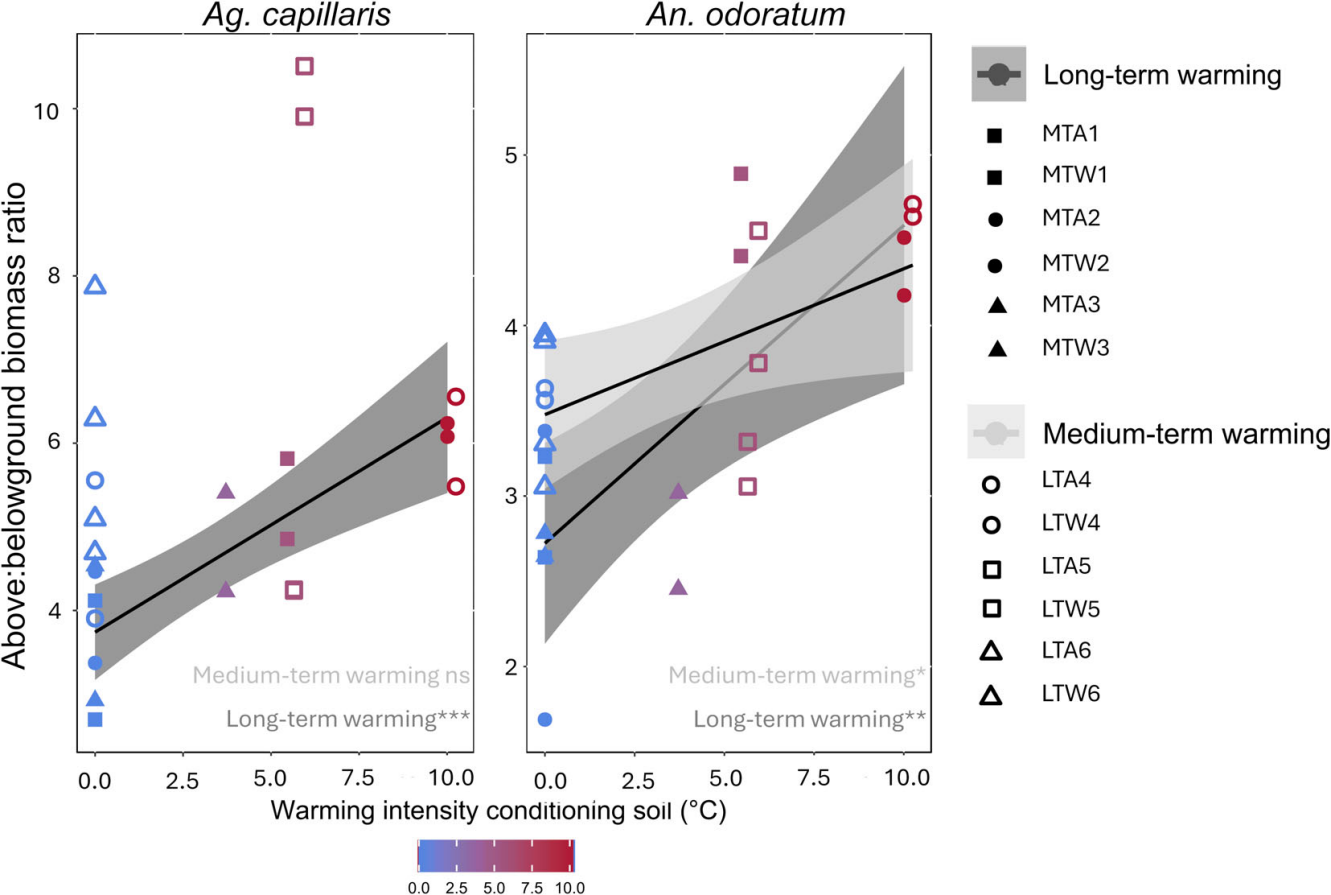
Aboveground biomass:belowground biomass ratio of *Ag. capillaris* (left) and *An. odoratum* (right) according to the warming intensity conditioning the soil inoculated. The long‐term warming intensity is indicated in dark grey and medium‐term in light grey. The shapes represent the origin of the different soils used: grassland with medium‐term (MT) or long‐term (LT) warming, conditioned by ambient (A) or warming (W). The numbers (1–6) indicate the transect. Asterisks indicate a significant effect of warming (*P < 0.05, **P < 0.01 and ***P < 0.001).

For both grass species, the aboveground biomass:belowground biomass ratio was positively correlated with the warming intensity conditioning the soil in LTW (R^2^ = 0.84, *P* = 0.0006 and R^2^ = 0.74; *P* = 0.006 respectively; Fig. 3). Under MTW, we observed a similar correlation in *An. odoratum* (R^2^ = 0.6, *P* = 0.04) but not in *Ag. capillaris* (R = 0.22; *P* = 0.48; Fig. 3).

**Fig. 4:**
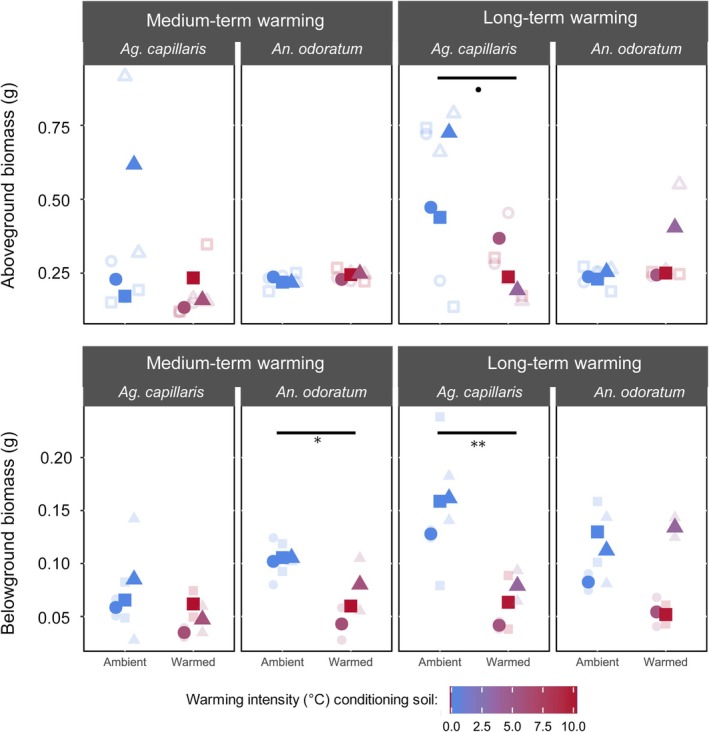
Aboveground (top) and belowground (bottom) dry biomass of *Ag. capillaris* and *An. odoratum* grown under drought conditions in soils conditioned by ambient temperature or warming (colour) from either medium term or long term. The bigger and fully coloured points are the averages of technical replicates that are transparent and smaller. Statistics were performed on averages of technical duplicates and only *P* values under 0.1 are shown (*P* < 0.1, *P < 0.05 and **P < 0.01). Shapes indicate that they are from the same transect.

Belowground biomass of both plant species was significantly reduced by the drought treatment (*P* < 0.0001; Fig. 4), yet for *An. odoratum*, this reduction depended on the warming treatment (*i.e*. ambient *versus* warmed; *P* = 0.05) as well as duration (*i.e*. MTW *versus* LTW; *P* = 0.03). Similarly to control conditions, *Ag. capillaris* grown under drought in LTW‐conditioned soils showed a lower belowground biomass than those grown in unwarmed ones. Interestingly, this reduction was of 59% on average (*P* = 0.004) under drought, while it was by only 44% under control conditions. We found a trend where *Ag. capillaris* aboveground biomass was lower (*P* = 0.06) when grown in soils originating from LTW soils compared to unwarmed soils that was not observed under control conditions (Fig. 4). *Ag. capillaris* was not affected by the microbiota resulting from MTW under drought, yet contrastingly, *An. odoratum* belowground biomass was reduced by MTW‐conditioned soils (*P* = 0.02), but we found no alteration of aboveground (*P* = 0.14) nor total biomass (*P* = 0.19).

### Plant‐associated fungal indicators

We found that the relative abundance of putative plant pathogens in inocula from LTW, that ranged from 0% to 4.5%, was consistently increased by warming, with the highest intensity (+10 °C) resulting in a near doubling in relative abundance compared to unwarmed (Table 1). However, this pattern was not observed under MTW, where the variation was higher, ranging from 0% to 12% (Table 1). Potential plant pathogens included fungal taxa from the *Ilyonectria*, *Itersonilia, Fusarium* and *Petrakia* genera (Table S2). The relative abundance of AMF taxa was low in unwarmed soils from LTW (0–0.25%; Table 1), but was consistently increased under LTW (0.3–0.85%; Table 1). No trend was however observed under MTW, where relative abundances were also low (0–1.35%; Table 1). Arbuscular mycorrhizal fungi ASVs included taxa from the *Claroideoglomus*, *Paraglomus*, *Diversispora* and *Glomus* genera (Table S3).

**Table 1:** Relative abundance of putative plant pathogenic fungi and arbuscular mycorrhizal fungi in unwarmed and warmed soil inocula under medium‐term warming (MTW) and long‐term warming (LTW).

	warming intensity	fungal putative plant pathogens		arbuscular mycorrhizal fungi	
		unwarmed	warmed	unwarmed	warmed
MTW1	+5.9 °C	11.97%	0.08%	1.05%	1.35%
MTW2	+10.2 °C	0%	4.35%	0.35%	0.10%
MTW3	+5.6 °C	2.58%	0%	0.05%	0%
LTW1	+5.5 °C	3.18%	4.53%	0%	0.85%
LTW2	+10 °C	1.92%	3.62%	0%	0.30%
LTW3	+3.7 °C	0.97%	1.32%	0.25%	0.55%

Increases in proportion with warming are indicated in green and decreases in orange.

## DISCUSSION

We observed that plants grown in soils inoculated with microbiomes originating from warmed sites displayed lower belowground biomass but unchanged aboveground biomass. The resulting increase in aboveground biomass:belowground biomass ratio was positively correlated with the warming intensity the soil microbiomes had been conditioned to. This response was however only in the case of LTW soils, which is coherent with a previous study conducted at the ForHot experimental site (Radujkovic *et al*. 2018), where warming was found to differentially shift the soil microbial communities, depending on the duration. At the ecosystem level, over 55 years of warming has led the grassland to a presumed new steady state (Walker *et al*. 2020), where the soil microbiome may result not only from innate variation in temperature sensitivity, but also from subsequent ecosystem changes that require longer times to take effect, such as through plants' responses. Additionally, some aspects of plant communities have shown stronger responses under LTW than under MTW, such as plant species loss (Walker *et al*. 2020). Moreover, indirect effects of warming on plant growth include a warming‐induced rapid and substantial loss in soil resources such as coupled N and C losses (Marañón‐jiménez *et al*. 2019) that is likely to increase demand for nutrient acquisition traits, possibly through the recruitment of beneficial microbes, for example, arbuscular mycorrhiza (Radujkovic *et al*. 2018), in accordance with the increase in AMF under warming we observed in the soil inocula.

In line with the findings of our greenhouse setting, Bhattarai *et al*. (2023) observed an increased aboveground biomass:belowground biomass ratio at the plant community level under LTW, but not MTW, under field conditions at the ForHot site. Similarly, they found an overall reduced fine root biomass under warming, but this was accompanied by a higher shoot biomass, which was hypothesised to result from increased litter. Here, we controlled for potential differences in soil chemistry, thus excluding the possible effect of an increased litter from previous seasons, yet observed a similar pattern, suggesting the decrease in root biomass may be at least partially microbially mediated. However, the higher biomass allocation towards shoots that was observed in the field may be a direct effect of warming enhancing plant metabolism, which would not be found in our study due to the standardised growth conditions. Moreover, and contrasting with our results, plant belowground biomass was already reduced under MTW in the field (Bhattarai *et al*. 2023), while microbiomes conditioned by MTW generally showed no effect on plant performance (except for under drought conditions). This suggests that apart from direct warming effects on plants, soil microbes may significantly contribute to this effect, and this contribution may be especially apparent over longer time frames. Interestingly, Zhou *et al*. (2022) found that warming generally increased biomass allocation to roots across biomes, but to a lower extent in wet environments and not in AMF‐associated plants, where belowground biomass remained unaltered by warming. This further supports the importance of AMF in the response of warming in such systems.

However, apart from beneficial plant‐associated microbes, soilborne plant pathogens can critically affect belowground biomass through infections. Here, we identified a consistent increase in putative fungal plant pathogens in LTW soils, which included taxa from the *Fusarium* genus, comprising some of the most important fungal plant pathogens with a wide range of diseases caused and hosts (Summerell 2019), or *Ilyonectria*, also an important soilborne plant pathogens of various herbaceous plant hosts (Lombard *et al*. 2014). The observed decreases in belowground biomass are most likely caused by increased adverse biotic interactions and to a lesser extent by an increased colonisation of roots by symbionts (*i.e*. mycorrhizal fungi) lowering the need for a more developed root system, or a combination of both. Warming can directly induce an increase in plant pathogen load (Liu *et al*. 2019; Delgado‐Baquerizo *et al*. 2020), or may indirectly favour plant pathogens by increasing soil nutrient availability, which is known to favour pathogens in grasslands (Lekberg *et al*. 2021).

While droughts seldom occur in the Icelandic grassland sites where our soil originated, their incidence is predicted to increase because of changing temporal precipitation regimes (Pfleiderer *et al*. 2019). Resistance to and recovery from drought are commonly regarded as general indicators of soil health (Joos *et al*. 2023). In line with our hypothesis, we found that the reduction in belowground biomass of plants grown with LTW‐conditioned microbiomes compared to those with unwarmed became even more pronounced under drought in the case of *Ag. capillaris*. Lau & Lennon (2012) showed that plants may form associations with soil microbial communities which are capable of quickly responding and adapting to environmental changes, hence profiting stressed plants. This could explain the exacerbated response of plants when grown in LTW‐conditioned microbiomes under drought: While these were primarily shaped to survive warming, those from unwarmed soils seem to have a higher capacity to respond to stresses, in a way that could favour plants (Angulo *et al*. 2022).

## CONCLUSION

While field experiments are necessary and provide more representative conditions, controlled experiments allow us to better understand mechanisms and disentangle complex interactions. Particularly, warming is expected to occur further, and understanding the mechanisms underlying plant responses to future climate is crucial. Root biomass tended to be reduced under warming‐conditioned soil microbiomes, and we identified both putative antagonistic and mutualistic taxa that may be contributing to such observations. Warming duration influenced these effects, suggesting that these warming‐induced changes in microbe‐mediated plant performance may take a long time to fully manifest and stabilise. This study therefore highlights the potential role of soil microbes on plant performance, which, if similar under field conditions, may lead to great consequences at the ecosystem level, such as plant productivity or nutrient cycling.

## AUTHOR CONTRIBUTIONS

CLNdC, EV, CDT and LCS designed the experiment with inputs from MC and JD, and CLNdC and LCS executed it. PS collected the soils from the field site managed by BDSCLNdC carried out laboratory analyses with help from LCSCLNdC analysed the data and wrote the first draft, which has been revised and improved by all co‐authors.

## FUNDING INFORMATION

This study was supported by FutureArctic, a European Union's Horizon 2020 framework programme for research and innovation under the Marie Skłodowska‐Curie grant agreement No. 813114.

## CONFLICT OF INTEREST

The authors declare no conflict of interest.

## Supporting information


**Method S1.** Field and laboratory processes for microbial community analyses.
**Table S1.** Temperature of unwarmed soils at sampling, and the average intensity of the warmed plots within the same transect.
**Table S2.** Taxonomy of the 15 most abundant fungal ASVs assigned as putative plant pathogens.
**Table S3.** Taxonomy of fungal ASVs from the Glomeromycota phylum, constituting AMF.

## Data Availability

Sequencing data used for this study will be made available in NCBI‐SRA database under the accession no. PRJNA1235632.
