# Supplementary material for: Soil microbiomes conditioned by long‐term warming affect plant belowground performance
Source: Plant Biol (Stuttg). 2026 Jan 19;28(4):1169–77. doi: 10.1111/plb.70182 (PMC13175946; doi:10.1111/plb.70182)
Supplement: Supplementary file 1 — Method S1. Field and laboratory processes for microbial community analyses. Table S1. Temperature of unwarmed soils at sampling, and the average intensity of the warmed plots within the same transect. Table S2. Taxonomy of the 15 most abundant fungal ASVs assigned as putative plant pathogens. Table S3. Taxonomy of fungal ASVs from the Glomeromycota phylum, constituting AMF. [file PLB-28-1169-s001.docx]

Supporting Information

**Article title**: Soil microbiomes conditioned by long-term warming affect plant belowground performance

**Authors**: Coline Le Noir de Carlan, Erik Verbruggen, Louna Colaert-Sentenac, Mathias Cougnon, Páll Sigurðsson, Bjarni D Sigurdsson, Jane Debode, Caroline De Tender

The following Supporting Information is available for this article:

Method S1 | Field and laboratory processes for microbial community analyses

**Table S1 |** Temperature of unwarmed soils at sampling, and the average intensity of the warmed plots within the same transect

**Table S2** | Taxonomy of the 15 most abundant fungal ASVs assigned as putative plant pathogens

Table S3 | Taxonomy of fungal ASVs from the Glomeromycota phylum, constituting AMF

Method S1 | Field and laboratory processes for microbial community analyses

In October 2019, before the winter season, as part of a related project (More information can be found in Metze *et al*., 2024), two types of cores were filled with local soil and installed into each plot. One, used as control, had a 1 mm mesh size allowing both roots and microorganisms in, and the other had a 30 µm mesh size, only allowing microorganisms, thus excluding roots. All cores are approximately 8.5 cm long with a diameter of ca 3.2 cm and were therefore installed in the upper layer of the soil. In each plot, four of each core type were installed in order to perform seasonal sampling, that later revealed a particularly low variation in microbial community composition over time. Core sampling occurred late June 2020, mid-August 2020, early-October 2020 and mid-April 2021, which the latter was used in the present study. During each sampling campaign, one of each type of core was collected per plot, and data originating from the control type (i.e. root-associated soil microbes) was used to explore putative plant-associated microbial taxa. The temperature was manually measured where each core was collected in triplicates. All soil samples were immediately placed on dry ice until they reached the laboratory where they were weighted for water content assessment and freeze dried.

**Library preparation**

DNA was extracted from approximately 0.2 g of dry soil using the DNeasy® PowerSoil® Kit (Qiagen, Hilden, Germany). Lower amounts of soil were used in case of failed extractions. The final elution volume was 60µL. Each DNA extract first went through a first round of PCR targeting the ITS and 18S regions. Primers used for ITS were ITS1f (CTTGGTCATTTAGAGGAAGTAA) and ITS2 (GCTGCGTTCTTCATCGATGC). Primers used for 18S were TAReuk454FWD1 (CCAGCA(G/C)C(C/T)GCGGTAATTCC) and TAReukREV3 (ACTTTCGTTCTTGAT(C/T)(A/G)A. All primers were amended with the NEXT label (Illumina Inc., USA). Each PCR mixture consisted of 16.3 µL of water; 5 µL of buffer; 0.5 µL of dNTP; 0.5 µL of each forward and reverse primer; 1 µL of BSA; 0.2 µL of Phusion DNA polymerase and 1 µL of template. For amplification of the 18S, each PCR mixture consisted of 15.3 µL of water; 5 µL of water; 0.5 µL of dNTP; 0.75 µL of each forward and reverse primer; 1 µL of BSA; 0.2 µL of Phusion DNA polymerase and 1.5 µL of template. For ITS: initial denaturation at 98°C for 60 secs; 35 cycles at 98°C for 30 secs; 55 °C for 30 secs and 72 °C for 30 secs followed by 72 °C for 10 mins.

PCR products for all genes were similarly diluted 50 times and subjected to a second PCR using barcoded primers with Illumina adapters at a concentration of 0.1 µM. Each PCR mixture consisted of 11.8 µL of water; 5 µL of buffer; 0.5 µL of dNTP; 0.2 µL of Phusion DNA polymerase and 5 µL of mixed index primers, and conditions were as follows: 98 °C for 60 secs followed by 12 cycles of 98 °C for 10 secs; 63 °C for 30 secs and 72 °C for 30 secs; and subsequently 72 °C for 5 mins. Obtained PCR products were run on an electrophoresis on a 1.5 % agarose gel at 95 V for 40 min for successful PCR amplification confirmation of all samples that were subsequently pooled into a single library, for ITS and 18S separately. They were subjected to a gel extraction using the NucleoSpin kit (Macherey-nagel, Germany). The library was quantified through real time PCR (Kapa Library Quantification Kits, Kapa Biosystems, Wilmington, USA), adjusted to a concentration of 4 pM and sequenced on a Illumina MiSeq platform (Illumina Inc., USA) using a 2 x 300 cycles paired-end sequencing. Another sequencing run was carried out using the same parameters, but modifying individual sample concentrations to get a satisfying and similar number of reads across samples.

**Sequence processing and taxonomy assignment**

Data was processed and analysed in R (v4.0.4). The raw sequences obtained from both sequencing runs were individually processed using the DADA2 inference algorithm (Callahan et al., 2016) to generate Amplicon Sequence Variants (ASV) for each marker gene separately. First, primers were removed using the *cutadapt* tool v4 (Martin, 2011). Reads were then quality filtered for 18S by truncating at 240 bp and 180 bp, and 250bp and 180bp for forward and reverse respectively, with a maximum expected error of 2 for both forward and reverse. For ITS, the maximum expected error was 3 and 5 for forward and reverse respectively. The *learnErrors* function was used to generate separate error models for forward sequences, which were then inferred (De Gruyter et al., 2021). Outputs from both runs were then merged using the *mergeSequenceTables* function and chimeras were then removed. Using the *assignTaxonomy* function18S ASVs were aligned to the PR2 database v4.72 (Guillou et al., 2013) and ITS ASVs were aligned to the UNITE database v8.3 (Abarenkov *et al.*, 2010) Furthermore, potential plant pathogens were identified using the FungalTrait database (Polme, 2020). Samples were rarefied to a sequencing depth of 6000 and 2000 for ITS and 18S respectively. We captured fungal sequences from both the ITS1 and the V4 region of 18S, and analysed AMF using data from 18S, after checking data from ITS1 showed similar trends.

Table S1 | Temperature of unwarmed soils at sampling, and the average intensity of the warmed plots within the same transect

|  | Temperature of unwarmed soils during sampling | Warming intensity of warmed soil compared to the temperature of the unwarmed plot of the same transect |
| --- | --- | --- |
| Medium-term warmed (MTW) 1 | 3 °C | + 5.9°C |
| Medium-term warmed (MTW) 2 | 0.8 °C | + 10.2°C |
| Medium-term warmed (MTW) 3 | 3 °C | + 5.6°C |
| Long-term warmed (LTW) 1 | 2 °C | + 5.5°C |
| Long-term warmed (LTW) 2 | 2.7 °C | + 10 °C |
| Long-term warmed (LTW) 3 | 2.4 °C | + 3.7 °C |

Table S2 | Taxonomy of the 15 most abundant fungal ASVs assigned as putative plant pathogens

|  | Phylum | Class | Order | Family | Genus | Species |
| --- | --- | --- | --- | --- | --- | --- |
| ASV_69 | Ascomycota | Sordariomycetes | Hypocreales | Nectriaceae | Ilyonectria | mors-panacis |
| ASV_217 | Basidiomycota | Tremellomycetes | Cystofilobasidiales | Mrakiaceae | Itersonilia | pannonica |
| ASV_827 | Basidiomycota | Tremellomycetes | Cystofilobasidiales | Mrakiaceae | Itersonilia | perplexans |
| ASV_224 | Ascomycota | Sordariomycetes | Hypocreales | Nectriaceae | Dactylonectria | estremocensis |
| ASV_165 | Ascomycota | Sordariomycetes | Hypocreales | Nectriaceae | Neonectria | lugdunensis |
| ASV_204 | Ascomycota | Dothideomycetes | Pleosporales | Melanommataceae | Petrakia | Unknown |
| ASV_526 | Ascomycota | Sordariomycetes | Hypocreales | Nectriaceae | Nectria | Unknown |
| ASV_1078 | Ascomycota | Dothideomycetes | Pleosporales | Didymellaceae | Epicoccum | dendrobii |
| ASV_265 | Ascomycota | Sordariomycetes | Hypocreales | Nectriaceae | Fusarium | salinense |
| ASV_829 | Basidiomycota | Agaricomycetes | Cantharellales | Ceratobasidiaceae | Rhizoctonia | fusispora |
| ASV_1641 | Basidiomycota | Tremellomycetes | Cystofilobasidiales | Mrakiaceae | Itersonilia | perplexans |
| ASV_364 | Ascomycota | Dothideomycetes | Pleosporales | Melanommataceae | Petrakia | Unknown |
| ASV_1128 | Ascomycota | Dothideomycetes | Venturiales | Venturiaceae | Protoventuria | alpina |
| ASV_1392 | Ascomycota | Sordariomycetes | Xylariales | Microdochiaceae | Microdochium | lycopodinum |
| ASV_1195 | Ascomycota | Dothideomycetes | Capnodiales | Mycosphaerellaceae | Mycosphaerella | tassiana |

Table S3 | Taxonomy of fungal ASVs from the Glomeromycotina phylum, constituting AMF

|  | Class | Order | Family | Genus | Species |
| --- | --- | --- | --- | --- | --- |
| ASV_8 | Glomeromycetes | Glomerales | Claroideoglomeraceae | Claroideoglomus | lamellosum |
| ASV_31 | Paraglomeromycetes | Paraglomerales | Paraglomeraceae | Paraglomus | Unknown |
| ASV_91 | Glomeromycetes | Glomerales | Claroideoglomeraceae | Claroideoglomus | lamellosum |
| ASV_104 | Glomeromycetes | Diversisporales | Diversisporaceae | Diversispora | Unknown |
| ASV_48 | Glomeromycetes | Glomerales | Glomeraceae | Glomus | versiforme |
| ASV_208 | Glomeromycetes | Glomerales | Glomeraceae | Glomus | Unknown |
| ASV_12 | Glomeromycetes | Glomerales | Glomeraceae | Glomus | macrocarpum |
| ASV_74 | Archaeosporomycetes | Archaeosporales | Ambisporaceae | Ambispora | Unknown |
| ASV_291 | Glomeromycetes | Glomerales | Glomeraceae | Glomus | Unknown |
| ASV_300 | Glomeromycetes | Glomerales | Glomeraceae | Glomus | Unknown |
| ASV_311 | Glomeromycetes | Gigasporales | Gigasporaceae | Scutellospora | calospora |

References

**Abarenkov K, Zirk A, Piirmann T, Pöhönen R, Ivanov F, Nilsson RH, Kõljalg U**. **2010**. The UNITE database for molecular identification of fungi – recent updates and future perspectives. *UNITE Community* **Version 10**.

**Callahan BJ, McMurdie PJ, Rosen MJ, Han AW, Johnson AJA, Holmes SP**. **2016**. DADA2: High-resolution sample inference from Illumina amplicon data. *Nature Methods* **13**: 581–583.

**De Gruyter J, Weedon JT, Elst EM, Geisen S, van der Heijden MGA, Verbruggen E**. **2021**. Arbuscular mycorrhizal inoculation and plant response strongly shape bacterial and eukaryotic soil community trajectories. *Soil Biology and Biochemistry* **165**: 108524.

**Guillou L, Bachar D, Audic S, Bass D, Berney C, Bittner L, Boutte C, Burgaud G, De Vargas C, Decelle J, *et al.*** **2013**. The Protist Ribosomal Reference database (PR2): A catalog of unicellular eukaryote Small Sub-Unit rRNA sequences with curated taxonomy. *Nucleic Acids Research* **41**: 597–604.

**Martin M**. **2011**. Cutadapt removes adapter sequences from high-throughput sequencing reads. *EMBnet* **17**: 10–12.

**Metze D, Schnecker J, Le Noir de Carlan C, Bhattarai B, Verbruggen E, Ostonen I, Janssens IA, Sigurdsson BD, Hausmann B, Kaiser C, *et al.*** **2024**. Soil warming increases the number of growing bacterial taxa but not their growth rates. *Science advances* **10**: eadk6295.

**Põlme S, Abarenkov K, Henrik Nilsson R, Lindahl BD, Clemmensen KE, Kauserud H, Nguyen N, Kjøller R, Bates ST, Baldrian P, *et al.*** **2020**. FungalTraits: a user-friendly traits database of fungi and fungus-like stramenopiles. *Fungal Diversity* **105**: 1–16.
